# A GaN-Based Wireless Monitoring System for High-Temperature Applications

**DOI:** 10.3390/s19081785

**Published:** 2019-04-14

**Authors:** Ahmad Hassan, Mohamed Ali, Aref Trigui, Yvon Savaria, Mohamad Sawan

**Affiliations:** 1 Department of Electrical Engineering, Polytechnique Montreal, QC H3T 1J4, Canada; mohamed.ali@polymtl.ca (M.A.); aref.trigui@gmail.com (A.T.); yvon.savaria@polymtl.ca (Y.S.); mohamad.sawan@polymtl.ca (M.S.); 2 Microelectronics Department, Electronics Research Institute, Cairo 12622, Egypt; 3 School of Engineering, Westlake University, Hangzhou 310024, China; 4 Institute of Advanced Study, Westlake Institute for Advanced Study, Hangzhou 310024, China

**Keywords:** high-temperature applications, wireless data transmission, GaN HEMT, integrated circuits, harsh environment

## Abstract

A fully-integrated data transmission system based on gallium nitride (GaN) high-electron-mobility transistor (HEMT) devices is proposed. This system targets high-temperature (HT) applications, especially those involving pressure and temperature sensors for aerospace in which the environmental temperature exceeds 350 °C. The presented system includes a front-end amplifying the sensed signal (gain of 50 V/V), followed by a novel analog-to-digital converter driving a modulator exploiting the load-shift keying technique. An oscillation frequency of 1.5 MHz is used to ensure a robust wireless transmission through metallic-based barriers. To retrieve the data, a new demodulator architecture based on digital circuits is proposed. A 1 V amplitude difference can be detected between a high-amplitude (data-on) and a low-amplitude (data-off) of the received modulated signal. Two high-voltage supply levels (+14 V and −14 V) are required to operate the circuits. The layout of the proposed system was completed in a chip occupying 10.8 mm^2^. The HT characterization and modeling of integrated GaN devices and passive components are performed to ensure the reliability of simulation results. The performance of the various proposed building blocks, as well as the whole system, have been validated by simulation over the projected wide operating temperature range (25–350 °C).

## 1. Introduction

Monitoring some critical parameters, such as temperature and pressure, in industrial applications is needed to control sensitive areas like combustion engines, gas turbines and oil wells. Placing sensors in harsh environments allows more accurate measurements to be made. However, it is still a very challenging task, especially at extremely high temperatures and pressures. In addition, many systems in industrial applications operate in multiple harsh environment situations, such as in combustion engines, where the high temperature is often directly proportional to the high pressure. Therefore, it becomes necessary to develop advanced harsh environment sensing technologies.

Silicon carbide (SiC)-based piezoresistive and capacitive sensors as well as polycrystalline diamond sensors are suitable for high-temperature (HT) and high-pressure (HP) measurements [1,2]. However, the output signal of these types of sensors is usually low and requires considerable embedded signal conditioning electronics. Platinum-based thermocouples are stable at extreme temperatures up to 2000 °C. However, their sensitivity is normally low and can be seriously affected by induced common-mode noise. Optical fiber sensing technology is a promising solution for HT and HP measurements. It is favorable because of the HT capability of optical fibers up to 1000 °C [3,4], in addition to their small size, light weight and resistance to electromagnetic interference (EMI). However, special housing for optical fibers is required at HT. This affects the applicability of optical sensors in several harsh environments.

Moreover, in many cases, sensors placed in harsh environments are frequently isolated from their control or power management units, which are usually located outside the harsh zone [5]. Commonly, these entities are interconnected with regular wires. However, these wired connections present many challenges in harsh environments for ensuring system light weight, simplicity and low-cost system implementation. In addition, drilling holes through the separation medium which is mostly metallic is not always an acceptable solution giving the potential risk of toxic chemical leakage, pressure or vacuum loss, and mechanical structure integrity weakening.

As a solution of this problematic, passive wireless sensing technologies in extreme environments have been investigated. Surface acoustic wave (SAW) sensors have been demonstrated at HTs up to 800 °C [6,7]. However, SAW sensors are very sensitive to the variation of material properties that is expected in harsh environments. Another possible solution is RF coupling sensors with a LC resonator circuit installed in the harsh environment [8]. The main drawback of RF coupling sensors is their limited HT capability, in addition to their sensitivity to EMI.

To address the aforementioned hindrances, a promising solution is to build an integrated microelectronic-based wireless transmission system. The transmission part of proposed integrated system is embedded along with the sensor interface into the harsh environment. The acquired information by the sensor interface is processed by the microelectronic system and wirelessly transmitted to an external receiver. However, when the high-environmental temperature exceeds around 150 °C–175 °C, traditional Si-based systems are no longer able to fit the requirements, due to the effective impact of HTs on the physical and electrical behavior of the technology used.

A possible solution is to provide cooling systems. However, such solutions introduce complexity in terms of added components that increase weight and size, which is often not acceptable, especially in critical applications such as deep-well drilling, automotive, and aerospace. Therefore, several influential corporations like Airbus, Safran, and Thales are seeking alternative microelectronic technologies that can work under HT conditions to develop wirelessly controlled systems that can harvest power from the surrounding HT and exchange data through wireless links [9,10,11].

Available semiconductors dedicated to HT applications, such as silicon on insulator (SOI), gallium arsenide (GaAs), and silicon germanium (SiGe) serve a relatively short range of temperature not exceeding 300 °C and for limited operation times [9,12,13,14], while the real industrial requirements can be much higher than these limits.

Wide bandgap (WBG) semiconductors are the main candidates in the foreseeable future to overcome the fundamental limits of available conventional electronics in HT applications [9,15]. Silicon carbide (SiC) and gallium nitride (GaN) are the best-known WBG devices that offer attractive features suitable for HT conditions. These features include wide bandgap (3 eV), high drift-saturation velocity, high thermal-conductivity, and low intrinsic carrier concentration [16].

Although GaN and SiC belong to the same WBG semiconductors family and share similar attractive properties, SiC has received a great deal of attention in the past decade, especially in the high temperature applications field. Several research results have been recently reported on developing SiC-based ICs for HT applications. For instance, multistage digital and analog SiC-ICs using 4H-SiC MESFETs are demonstrated in [17] showing correct operation at 300 °C. Seventeen circuits implemented with the Raytheon’s 4H-type HTSIC process were reported in [18] and successfully tested at 300 °C. In [19], HT voltage and current references are designed with a silicon carbide CMOS process. Their operation and stability were reported in the 25 °C to 540 °C temperature range. Using 6H-SiC depletion-mode JFET transistors, the design characterization of various logic circuits (inverter, NAND, and NOR) were reported in [20] at extreme temperatures reaching 550 °C. In [21], low-voltage 4H-SiC n-p-n bipolar devices are used to implement OR–NOR gates and a three-stage ring oscillator. The integrated circuits have been successfully tested up to 300 °C. Based on 6H-SiC n-channel depletion-mode JFETs, differential amplifiers were successfully fabricated for use in HT differential sensing [22]. The reported circuits were characterized at temperatures reaching 450 °C. A simple analog amplifier and a NOT logic gate reported in [23] were fabricated using 6H-SiC JFET technology and were successfully operated for thousands of hours at 500 °C. The authors in [24] report the first analog-to-digital converter (ADC) implemented using SiC along with the first CMOS digital-to-analog converter (DAC). These circuits were tested in the 25 °C to 400 °C temperature range.

Although remarkable advances have been achieved, the development of SiC-based systems dedicated for sensing applications is still in its early beginnings. Indeed, the majority of the reported SiC ICs are realized with either a small number of implemented devices or on large integrated areas having low device integration density. There are still many obstacles limiting widespread adoption of SiC sensing systems, including immature foundry processes, design kits and device models. Only in [25,26] of integrated SiC-based wireless sensing systems were reported for temperature and pressure measurements respectively. The developed RF transmitters were successfully demonstrated from 25 °C to 450 °C. However, the proposed system represents only the RF transmitter part, where the receiver is a Tektronix RSA3303B real-time spectrum analyzer. In addition, the EMI effect on the wireless transmission performance was not investigated in spite of the expected metallic environment between the transmitter and receiver.

On the other hand, III-Nitride technologies, primarily GaN, exhibit substantial performance improvements over the other semiconductors with respect to response speed and operating temperature limits [20], in addition to the temperature stability of electron concentration in the HEMT channel that makes GaN devices more stable over wide temperature ranges. Despite considerable efforts to develop GaN devices operating at HT above 600 °C [27], 800 °C [28], 900 °C [29] and 1000 °C [30], few research projects are directed toward the development of integrated microelectronic circuits and systems based on GaN devices.

To the best of our knowledge, none of the previously reported results relate to system level design or implementation. The reported works only show the implementation of simple circuits like inverters, comparators, ring oscillators, and one stage differential amplifiers [31,32,33]. In [31], a GaN-based enhancement/depletion (E/D)-mode inverter was successfully fabricated and tested from room temperature to 300 °C. GaN heterojunction FET (HFET) depletion mode devices are used in [32] to implement various circuits: Not gate, comparator, ring-oscillator and frequency divider. A 31-stage ring-oscillator was tested at temperatures reaching 265 °C and it was shown that this circuit returns to the original performance after returning to room temperature. A novel AlInN/GaN-based IC was demonstrated in [33]. The fabricated ICs comprised an inverter and a differential amplifier that showed stable performance up to 500 °C.

In this paper, we present the first complete design of a wireless data transmission system based on GaN500 technology that is intended to acquire signals from sensors installed in HT environments and transmit the data through an inductive link to an external receiver. We focus on the electronic part, including the transmitter and the receiver, rather than on the sensor interface itself. The main goal is to implement a generic integrated system that could be utilized in different types of harsh environment sensors, including pressure and temperature sensors, with minor adaptation. Section 2 includes the description of the proposed system. The HT modeling and characterization of GaN devices and passive elements are presented in Section 3. The main considerations about the adopted GaN technology and the circuit building blocks are described in Section 4, along with corresponding simulation results. Conclusions are the subject of Section 5.

## 2. Proposed Wireless System

Typical monitoring sensors in HT applications require two types of connections; a power source coming from an external power supply and the data connection to send the data monitored by the sensor interface to the external system. In addition, most harsh environment sensors are installed inside compact metallic capsules. For this reason, different techniques have been adapted for transmission through metallic barriers [5] for example RFID [34], planar IPT [35] and piezoelectric ultrasound [36]. However, these approaches are limited due to the low-transfer efficiency and are restricted to specific applications (soft and thin metals).

Furthermore, harsh environment conditions intensively affect the curie point of piezoelectric materials at which their functionality is lost. In addition, the system performance of ultrasound techniques is highly reliant on the coupling quality and requires a direct grounding through the metal wall. A weak coupling leads to significant impedance mismatch over the acoustic-electric channel and causes a direct drop in the power-transfer efficiency and transmission data rate. Moreover, an inductive power transfer (IPT) system is rarely used to transfer power through a metallic medium, due to the high electrical conductivity that induces high Eddy current losses, and also due to the high magnetic permeability of metal walls, which form a shielding zone for inductive fields.

However, in our previous work [11], we described the design of a robust inductive link that can wirelessly transmit power to pressure and temperature sensors installed in harsh environments. The link was designed to operate at temperatures up to 500 °C and pressure differential of up to 100 Bar. Different materials were evaluated, including titanium and steel, and their properties were investigated.

**Figure 1:**
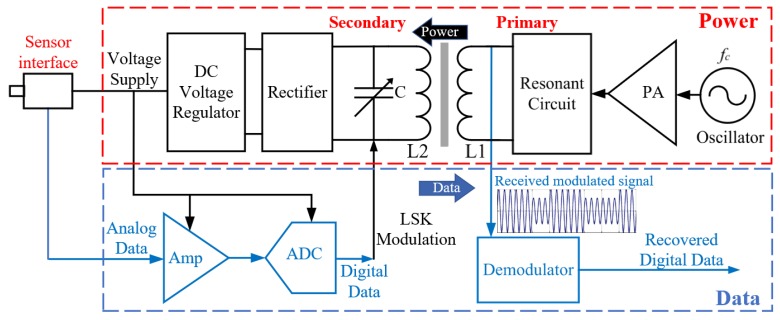
Block diagram of the proposed power and data transmission system.

Figure 1 shows the proposed harsh environment wireless power and data communication system based on an inductive link to transmit power and data through a metallic barrier. The work presented in this paper deals only with the data path linking a low-amplitude analog signal, provided by a sensor interface (on the secondary side), to the delivered data on the primary side. The amplified signal is then applied to an ADC to provide a digital signal that can modulate the power signal coming from the primary inductive coil using load shift keying (LSK). On the primary side, the demodulator block detects the modulated signal and recovers the transmitted digital data.

Load-shift keying modulation is a technique that produces a variation in a secondary circuit impedance that is reflected as a variable impedance in a primary circuit. This impedance change allows recovering digital data that is virtually transmitted from one side to the other side of the inductive link by sensing the reflected impedance variations. In our system, the inductive link used to deliver power from an external system is the same path that can be utilized to send back the data coming from the sensor. Therefore, LSK modulation is used to ensure simplicity of the wireless path implemented using a single inductive link providing power and data transmission.

## 3. Modeling and Characterization:

The technology adopted to implement the proposed GaN-based data communication system is the GaN500 HEMT provided by the Canadian Photonics Fabrication Center (CPFC) of the National Research Council of Canada (NRC). This technology is fabricated on 3-inch silicon carbide (SiC) wafers of 75 µm thickness. It features 0.5 µm long metal gates, two metal layers (1ME and 2ME) for interconnections, 50 Ω/sq nichrome resistors, and MIM capacitors (0.19 fF/µm^2^). The SiC substrate offers high-thermal conductivity and small lattice mismatch to a GaN layer, which is compatible with high-temperature applications. The transistors are field-plated designs that exhibit much lower gate leakage current and higher breakdown voltage than non-field plated devices.

The design and simulation of the proposed wireless system are completed under the Keysight’s Advanced Design System (ADS) tool using the NRC Gallium Nitride MMIC Foundry Design kit (GaN500v3.10). This kit provides a transistor based on Angelov model that integrates temperature effects and can be used to simulate the impact of internal self-heating and external ambient temperature on the behavior of simulated circuits. However, in this GaN500v3.10 design kit, the Angelov model of GaN500 device was not validated at temperature higher than 200 °C and the passive components were assumed to have values constant with temperature. Therefore, we performed the HT characterization of GaN500 devices and passive elements to validate the Angelov model at HT and we developed an accurate model of passive elements that enables simulating the temperature effects on the proposed design.

### 3.1. GaN500 HEMT

**Figure 2:**
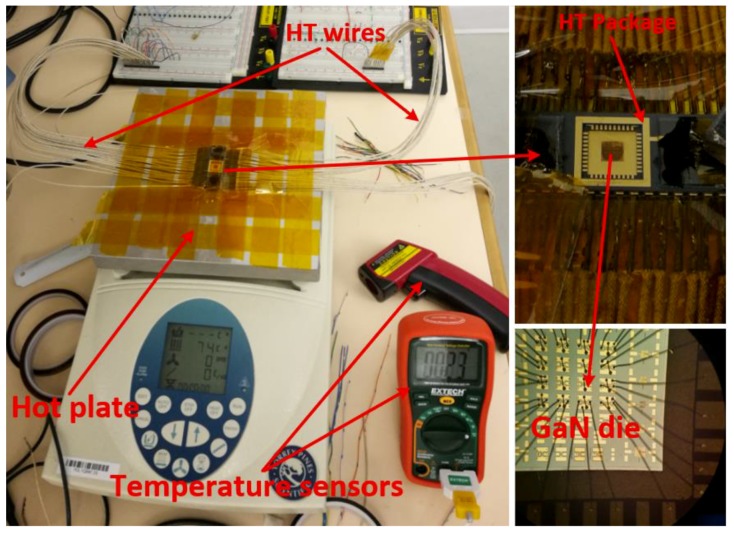
Experimental setup for HT GaN500 device characterization.

To ensure the validity of the available transistor model over the desired temperature range, we performed the HT I-V characterization of several fabricated GaN500 devices at various temperatures ranging from 25 °C to 350 °C. The experimental setup that was used is depicted in Figure 2 that shows GaN500 devices wire-bonded to the pads of a ceramic HT package and HT wires that are used to connect the package to the measurement devices.

High-temperature experiments were performed using a hot plate providing temperatures controllable over time. Temperatures were cycled between room temperature (25 °C) and 350 °C. An infrared thermometer and a direct-contact thermocouple were utilized to measure the operating temperature of the tested die surface that was used in combination with the temperature displayed on the hot plate control.

**Figure 3:**
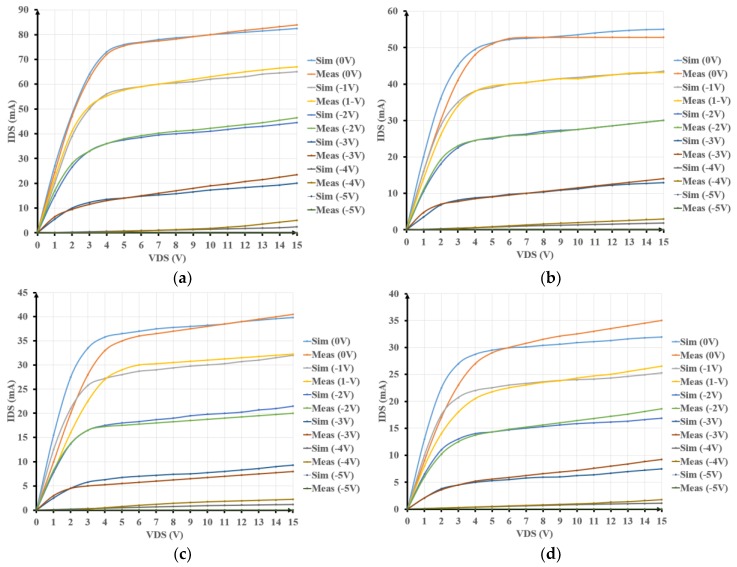
Measured and simulated I-V characteristics of a GaN500 device at: (**a**) 25 °C; (**b**) 200 °C; (**c**) 300 °C; (**d**) 350 °C.

The output I-V characteristics of a typical GaN500 transistor (L_Gate_ = 500 nm and W_Gate_ = 50 µm) are depicted in Figure 3. Each graph reports measured and simulated I-V characteristics for each of the following temperatures: (a) 25 °C, (b) 200 °C, (c) 300 °C, and (d) 350 °C. Each graph plots curves for each of six voltage levels, from −5 V (switch-off voltage) to 0 V (maximum switch-on voltage) applied to the device gate (V_GS_). The different curves are obtained by sweeping the drain-source voltage (V_DS_) from 0 V until 15 V. The reported results show the good match obtained between simulated and measured values for a given transistor aspect ratio and applied temperature. These results confirm the suitability of Angelov’s model over a 25 °C to 350 °C temperature range. The saturation current I_DS_ at V_GS_ = 0 V drops from 80 mA at T = 25 °C to 33 mA at T = 350 °C. This is a 40% drop of I_DS_ at V_GS_ = 0 V between 25 °C and 350 °C. Very similar current drops are obtained for the other I_DS_ at different V_GS_ levels.

### 3.2. Integrated Passive Components

**Figure 4:**
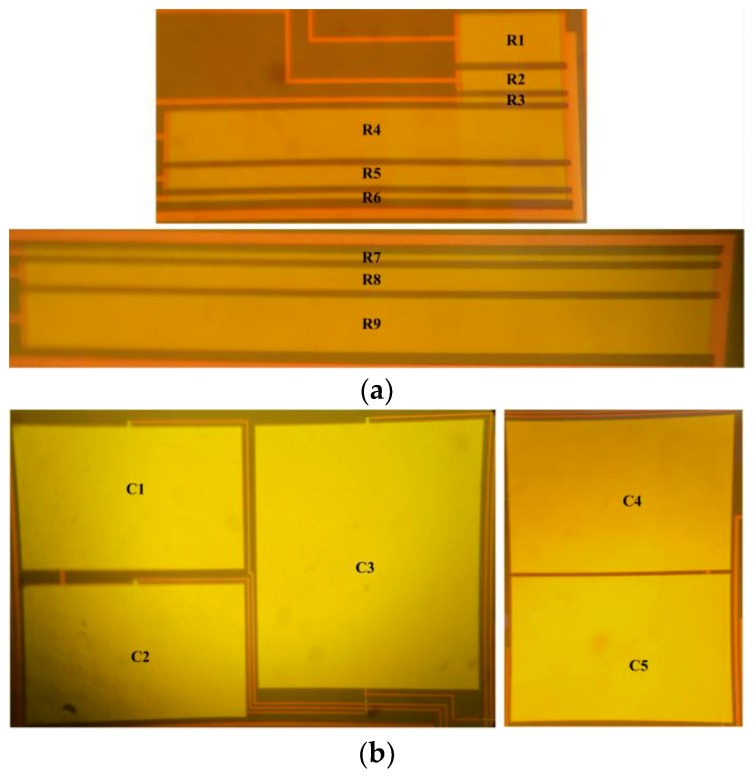
Integrated passive components for HT characterization: (**a**) Resistors; (**b**) Capacitors.

As shown in Figure 4, the process allows integrating compact passive devices, but provides no model of their sensitivity to temperature. As we intend to operate circuits reliably over a wide temperature range, the sensitivity of various passive components was characterized. Therefore, resistors (R1 to R9) and capacitors (C1 to C5) of various sizes (see Table 1) were fabricated as shown in Figure 4. The same experimental setup shown in Figure 2 was used to perform their HT characterization over the 25 °C to 350 ^o^C temperature range.

**Figure 5:**
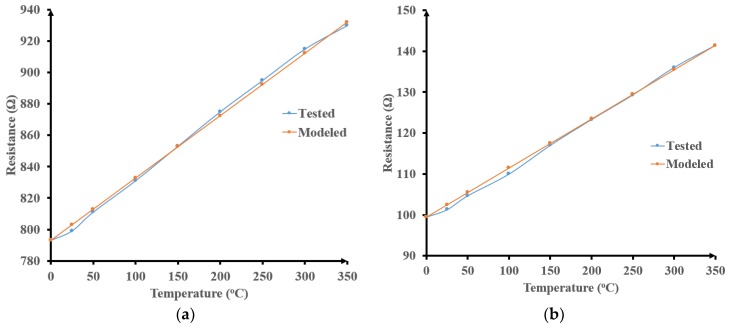

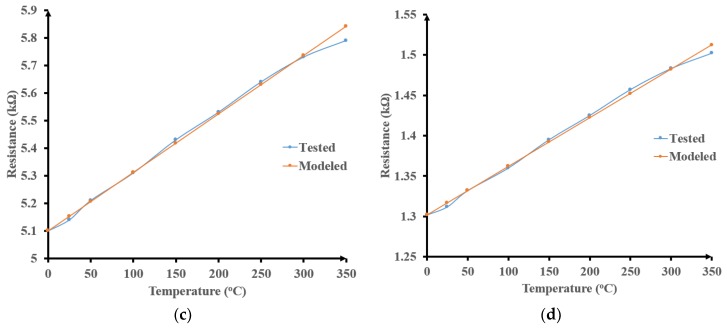
HT characterization of different integrated resistors: (**a**) R1; (**b**) R3; (**c**) R7; (**d**) R8.

The measured resistances of R(1,3,7,8), as shown in Figure 5, linearly increase with temperature. From the collected data, Equation (1) was extracted:

R_t_ = R_0_ (1 + 4 10^−4^ T) + 0.08 T (1)

It predicts the new resistance (R_t_) at any temperature (T) between 0 °C and 350 °C from its value at room temperature (R_0_). The resistance values predicted with equation (1) are plotted in Figure 5 which allows comparing them with the experimental results. This confirms the accuracy of the proposed model over the explored wide temperature range for all resistors. This predicted temperature variation of resistor values is included in all wireless monitoring system models.

**Figure 6:**
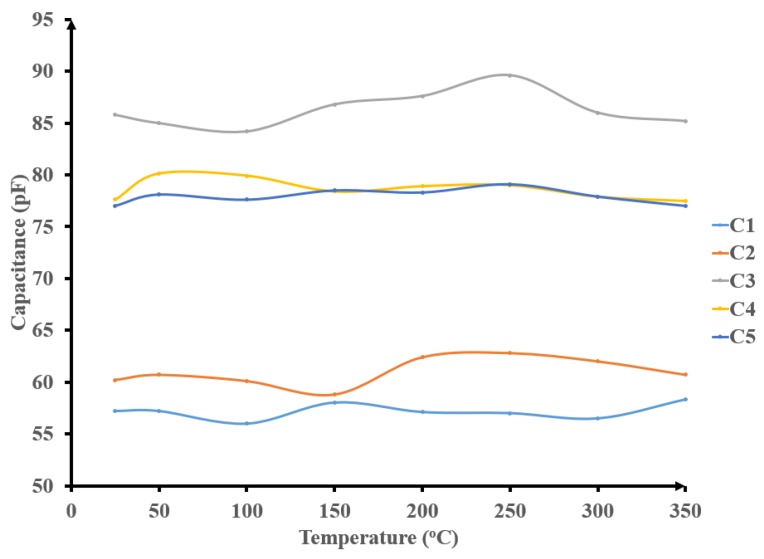
HT characterization of different integrated capacitors.

The stability of the capacitors was characterized with an impedance analyzer (Agilent 4294A). Figure 6 shows the capacitance of three different capacitors (C1 = C2 and C4 = C5) over the 25 °C to 350 °C temperature range. The results show the stable values observed for each capacitor with minor variations observed due to the added variable testing probe parasitic capacitance. Therefore, the capacitors in our design are considered to have constant values with temperature.

## 4. Circuit Design and Simulation Results

After we confirmed the validity of the GaN500 Angelov model available in the design kit and the passive elements model at HT up to 350 °C, we used these devices and passive elements to design the proposed wireless transmission system (including modulator and demodulator) and corresponding building block circuits taking into consideration the impact of temperature on the circuits and systems behavior.

However, since there is only one transistor type available in the GaN500 kit, implementing a complete modulation/demodulation system became a challenging task. First, we reviewed the applicable circuit design techniques used with WBG normally-on devices [20,33]. The available GaN500 depletion mode normally-on transistor requires V_GS_ = −5 V to completely switch off its conductive channel. Therefore, a negative input logic level is needed to drive the various circuits and consequently it sets a need to match the input-output controlling signals. Level shifters were added to the logic circuits to solve the incompatibility between the input and output levels and three supply voltage levels are needed (V_DD_ = +14 V, V_SS_ = −14 V, and Gnd).

Another challenging condition is the HT (350 °C) operating environment that the system should endure reliably while monitoring various sensors. Those two hindrances reduce the possibilities of adopting complex design techniques and are a strong motivation to reduce the number of transistors used to implement the various system building blocks.

**Figure 7:**
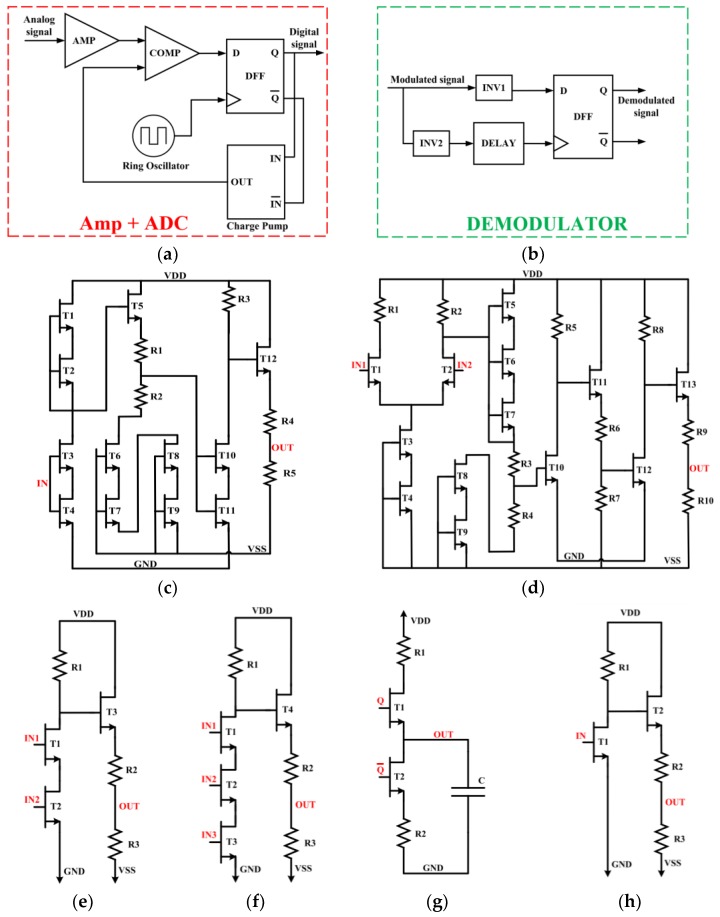
Data transmission system: (**a**) Block diagram of the transmitter; (**b**) Block diagram of the receiver; (**c**) Schematic of the amplifier; (**d**) Circuit diagram of the comparator; (**e**) Schematic of a 2-input NAND gate; (**f**) Schematic of a 3-input NAND gate; (**g**) Charge pump circuit; (**h**) Schematic of an inverter.

Figure 7 presents the block diagram of the proposed data transmission system, as well as the circuit implementation of its building blocks. The main goal of this work is to implement a generic integrated system that could be utilized in different types of harsh environment sensors such as HT and HP sensors. The proposed system could be adapted to fit the specific requirements of many types of sensors. To validate our system concept, a possible example is a pressure sensor [37]. It is a piezoresistive MEMS pressure sensor from Kulite Semiconductor Products, Inc. (Model No. XTEH-10L–190L). This sensor is a static pressure transducer with rated temperature of 500 °C (only for the sensor head) and rated pressure up to 210 Bar. It typically produces a small amplitude signal ranging between 0 and 100mV when the measured pressure ranges from 0 to 210 Bar.

### 4.1. Front-End Amplifier

**Figure 8:**
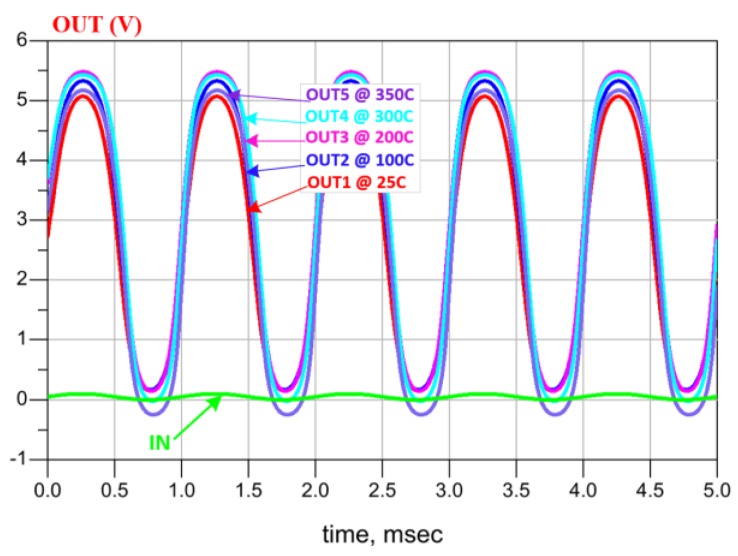
Amplifier simulated results at different temperatures.

A front-end amplifier block is used in the data transmission path as shown in Figure 7c. Its input signal is provided by the pressure sensor with minimum and maximum voltage of 0 V and 100 mV respectively. To amplify that signal, a 50 V/V two-stage amplifier architecture was adopted. Each stage of this amplifier comprises a level shifter to maintain the input-output compatibility. Simulation results, reported in Figure 8, show a sinusoidal input signal (IN = 0, +100 mV) and the corresponding stable amplified output signal (OUT = 0, +5 V) over the wide temperature range of 25 °C to 350 °C.

### 4.2. Analog to Digital Converter (ADC)

The amplified signal obtained from the pressure sensor is applied to an ADC. The block diagram of this ADC is presented in Figure 7a. The proposed ADC is based on the delta modulation technique. Its architecture is optimized to reduce complexity while consuming little power and area. The analog input signal, coming from the amplifier, is compared with the analog feedback signal produced by the charge pump to provide a digital output applied to a D-Flip Flop. Note that the charge pump in Figure 7g includes an output capacitor converting current pulses into an analog value that can be compared with the amplified analog signal coming from the sensor. The latter is used to synchronize the extracted digital signal by the clock signal coming from a ring oscillator to provide the final digital modulating waveform. The latter waveform is used along with its complementary value to drive a charge pump. The output data from the ADC is employed to drive the LSK modulator.

**Figure 9:**
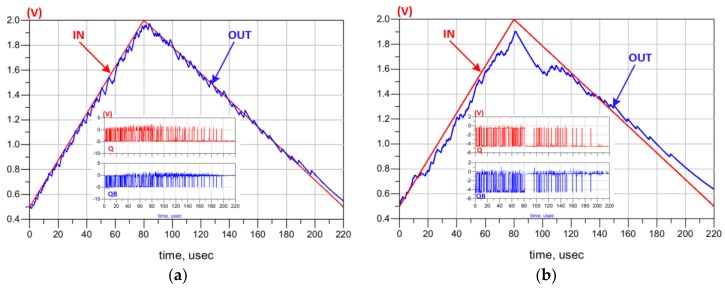
Simulation results of the data transmission chain with the input in red and the output in blue at: (**a**) 25 °C; (**b**) 350 °C. The recovered Q an QB streams are plotted in the embedded graphs.

The simulation results at 25 °C are shown in Figure 9a. Note that the feedback signal (OUT), coming from the charge pump, follows the amplified analog input signal (IN), where IN presents the monitored analog signal of the pressure sensor after amplification. Q and QB (plot embedded in Figure 9a) are used to control the direction of the charge pump current. At 350 °C, the ADC is still working, as shown in Figure 9b, despite the observable impact of the HT on its performance. The remaining of this section describes the main circuits composing the ADC as well as the simulation results confirming their functionality.

#### 4.2.1. Comparator

**Figure 10:**
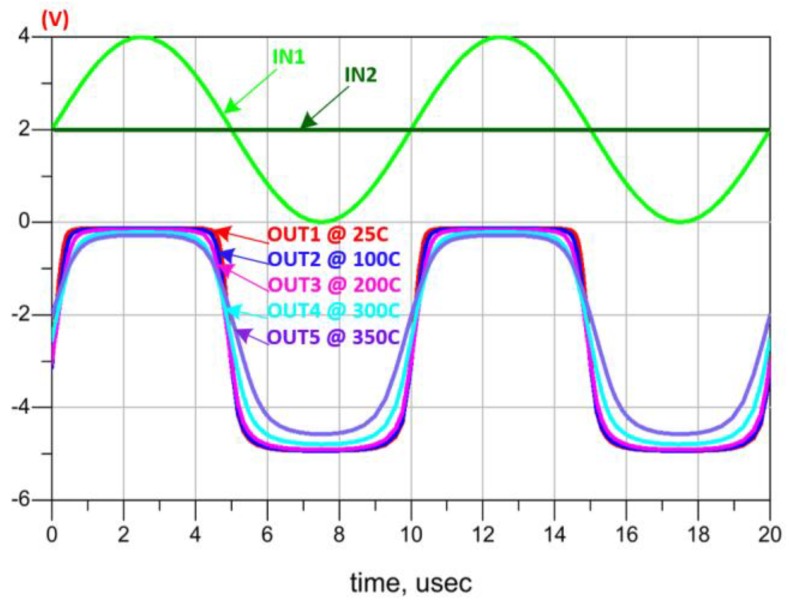
Comparator simulations at different temperatures.

Figure 7d presents the schematic design of the comparator where a first stage differential amplifier is followed by a series of adapted inverters and level shifters to reach the desired comparison accuracy. Figure 10 shows the comparator output and its good performance over the 25 °C to 350 °C temperature range.

#### 4.2.2. D-Flip Flop

**Figure 11:**
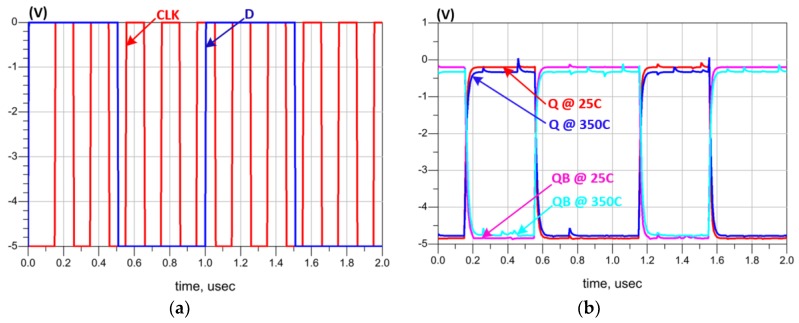
D-Flip Flop simulation results: (**a**) Input signals; (**b**) Output Data.

The D-Flip Flop design is based on five 2-input and one 3-input NAND logic gates where the corresponding schematic design is shown in Figure 7e,f, respectively. The simulation results, shown in Figure 11, confirm the good operation of the D-Flip Flop even at 350 °C.

#### 4.2.3. Charge Pump

**Figure 12:**
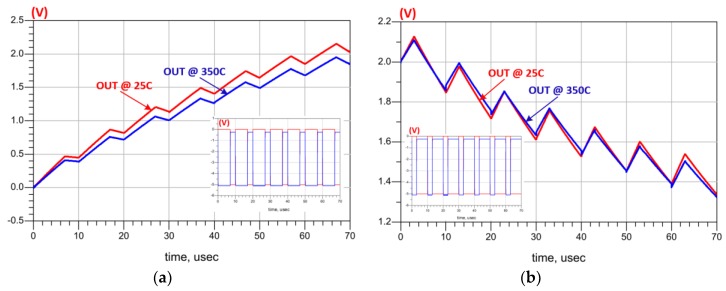
Impact of the temperature on Charge pump during: (**a**) Charging; (**b**) Discharging.

As briefly mentioned before, the purpose of the charge pump circuit is to convert its digital input stream into an analog output (DAC). It is required by the ADC to provide an analog feedback signal to be compared with the initial input analog signal and minimize the error rate of the modulation process. For our specific application (pressure sensor), the charge pump is designed to provide an output with minimum voltage (Vmin = 0 V) and a maximum voltage (Vmax = 2 V) to fit into the proposed ADC system. The schematic of the charge pump circuit is shown in Figure 7g where two complementary inputs (Q and QB) are needed to charge and discharge the capacitor (C). The proposed charge pump shows a correct behavior at 25 °C and 350 °C during charging and discharging as depicted in Figure 12a,b, respectively.

#### 4.2.4. Ring Oscillator

**Figure 13:**
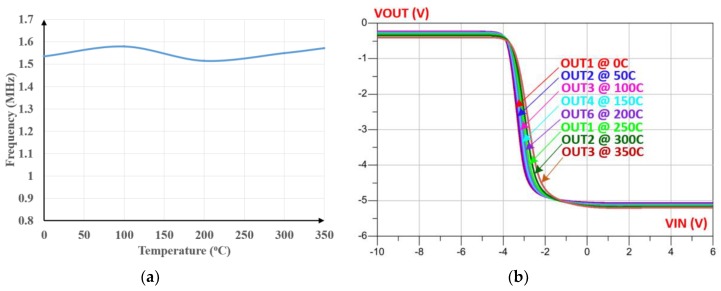
Impact of the temperature on: (**a**) Oscillation frequency; (**b**) voltage transfer characteristics of the inverter.

A 7-stage ring oscillator is implemented to generate a 1.5 MHz output signal followed by an inverter to sharpen the generated waveform. This frequency value is appropriate with the specific requirements of wireless inductive link through metallic barrier where a low-frequency signal is highly recommended [3]. Figure 13a shows the variation of the oscillation frequency as a function of temperature. Note that the maximum deviation of the frequency is around 4.2% over the considered wide temperature range of 25 °C to 350 °C.

#### 4.2.5. Inverter

The schematic of the inverter that was used is shown in Figure 7h. This design must be modified in terms of transistors size, resistor values and sometimes by adding parallel capacitors to the output based on design requirements. The simulated voltage transfer characteristics of a typical inverter are depicted in Figure 13b, confirming its correct operation over the whole range of temperatures considered.

### 4.3. Proposed Demodulation System

The block diagram of the proposed demodulator is presented in Figure 7b. The demodulator receives the LSK modulated signal (where there are two different amplitudes, high-level for data-on and low-level for data-off) and recovers the initial data.

The novel idea here is to use an inverter (INV1) which is carefully designed to detect only the high amplitude voltage (data-on) of input signal. On the other hand, INV2 is used to switch continuously regardless of the input voltage signal amplitude to generate the clock signal of the D-Flip Flop. This signal is delayed by the average of the DELAY block to ensure matching with the detected data signal. Both data-on and data-off amplitude voltage are recommended to be as close to each other as possible to ensure higher power transfer efficiency during LSK modulation.

The minimum amplitude of the data-on signal that can be detected by INV1 is ±5 V. In other words, to reach a higher power transfer efficiency, the amplitude of the data-on signal could be increased to more than ±5 V, as much as needed, until reaching the required power transfer efficiency, without applying any change on the proposed demodulator. In parallel, the maximum amplitude of the data-off signal is ±4 V and it could be less than that, but it would reduce the power transfer efficiency. Therefore, to validate the concept, the modulated signal (IN) is selected with a high-amplitude of ±5 V (minimum data-on) and low-amplitude of ±4 V (maximum data-off) to show the performance of modulators in the critical case. Consequently, a 1.0V amplitude difference (20% of the maximum amplitude) is reached between the high-amplitude (data-on) and the low-amplitude (data-off) of the modulated signal (IN) with the proposed demodulator.

**Figure 14:**
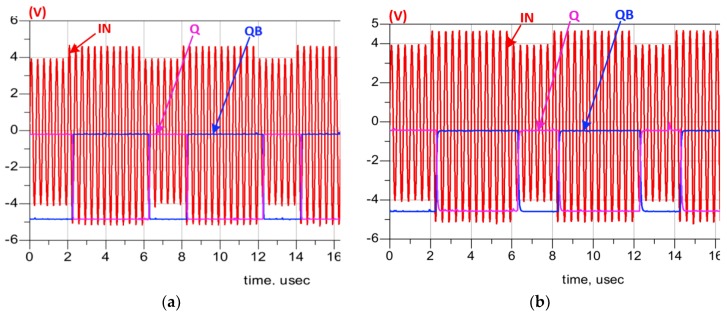
Demodulator simulation results of input/output signals at: (**a**) 25 °C; (**b**) 350 °C.

**Figure 15:**
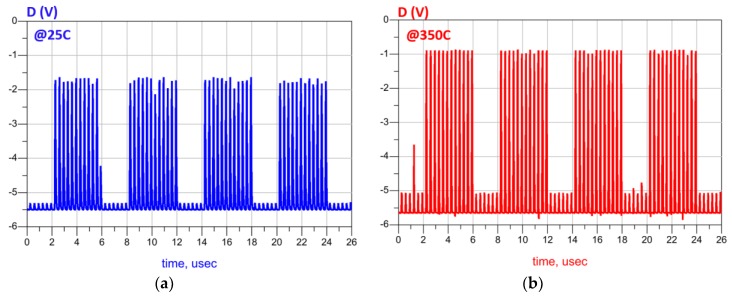
Impact of the temperature on detected data (D) at: (**a**) 25 °C; (**b**) 350 °C.

**Figure 16:**
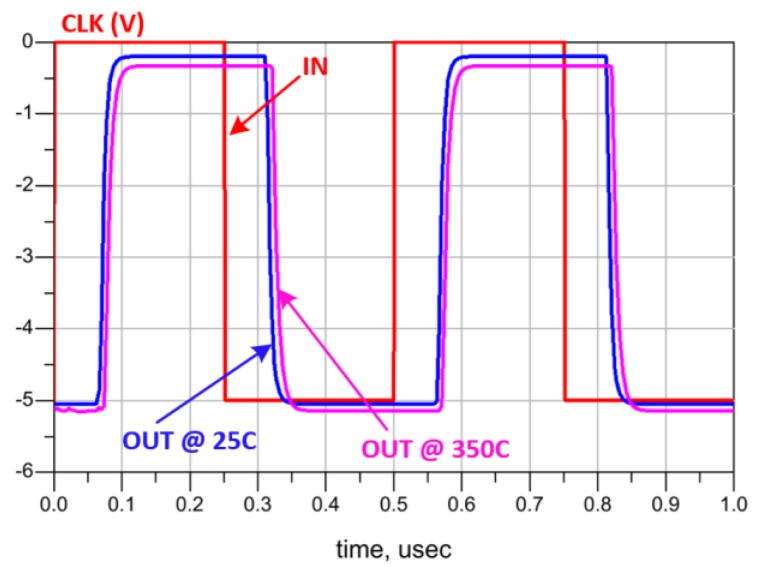
Temperature impact on DELAY circuit.

The retrieved demodulated signal (Q) and its complementary (QB) at 25 °C are shown in Figure 14a. INV1 detects only the high-level (± 5 V) pulses of IN and ignores the low-level (±4 V) ones as seen in Figure 15a. The required time delay to shift the clock (CLK) and ensure its intersection with the detected data (D) is comparatively large due to the low operating frequency. Therefore, a long chain of inverters (20 stages) is utilized to design the DELAY block. At 350 °C, the demodulator shows an excellent stability as seen in Figure 14b and Figure 15b. In Figure 16, the simulation results confirm the robustness of the designed DELAY block that shows a slight increase (10.5%) of its time delay at 350 °C.

**Figure 17:**
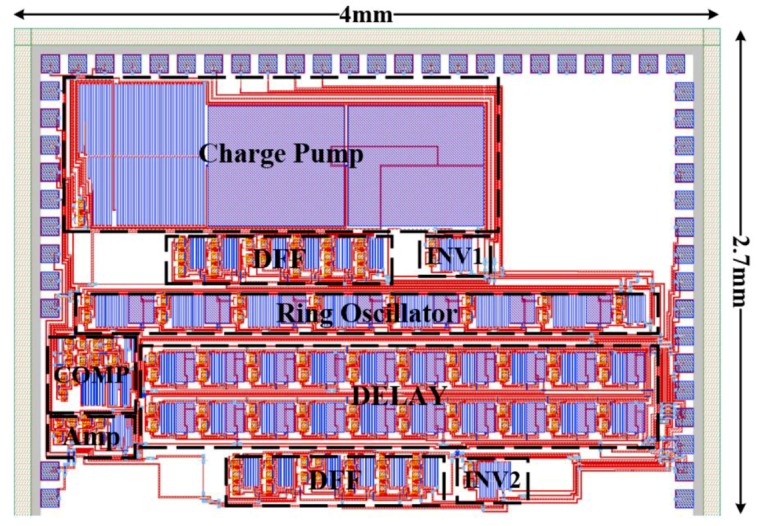
Chip layout view of the proposed data transmission system.

A complete chip layout (Figure 17) for the proposed system was performed. The overall area of the chip is 4.0 mm × 2.7 mm. The power consumption and the occupied area of the ADC, demodulator and their corresponding building blocks are listed in Table 2. It is remarkable that the HT has a positive impact on power consumption, with power consumption reduced by more than 40% at 350 °C for some blocks. The power consumption and area of the digital demodulator are dominated by the DELAY block, which should be optimized in future work.

## 5. Conclusions and Future Work

We described novel circuit techniques intended for implementing wireless data transmission systems dedicated for high-temperature (HT) applications. The introduced system is integrated using AlGaN/GaN HEMT devices to benefit from its corresponding outstanding properties, commercial availability, and ability to endure the HT environment. The HT characterization of GaN500 devices, integrated resistors, and integrated capacitors was performed to validate their corresponding models used in system simulations. The design considerations of the proposed system were discussed taking into account the specific requirements of the targeted applications and the limitations of adopted technology. Circuit simulations of subsystems and building blocks were performed over the 25 °C to 350 °C wide temperature range to validate the capability of the GaN technology to implement an integrated wireless system that can be used in harsh environment applications. Future work should focus on circuit design optimization to further reduce area and power consumption.

## Figures and Tables

**Table 1:** Integrated Resistors and Capacitors.

Resistor	W/L (µm)	Value @25 °C (Ω)	Capacitor	W/L (µm)	Value @25 °C (pF)
R1	50/100	103	C1	500/315	57
R2	20/100	220	C2	500/315	60
R3	5/100	805	C3	500/600	86
R4	50/400	340	C4	400/600	77
R5	20/400	815	C5	400/600	77
R6	5/400	3150			
R7	5/650	5150			
R8	20/650	1320			
R9	50/650	540			

**Table 2:** Power consumption and area of the various system building blocks.

Circuit	Power (mW) @ 25 °C	Power (mW) @ 350 °C	Area (mm^2^)
ADC	1100	720	3.2
Amp	658	308	0.45 × 0.2
COMP	770	420	0.42 × 0.46
D-FLIP FLOP	126	108	1.2 × 0.25
Ring Oscillator	210	189	3.2 × 0.22
Charge pump	1.4	1.2	2.4 × 0.8
Demodulator	4438	4000	2.01
INV1	32	28	0.27 × 0.21
INV2	91	70	0.26 × 0.23
DELAY	3318	3010	2.9 × 0.55
